# The presence of mahi-mahi at multi-predator feeding aggregations modifies striped marlin attack behaviour

**DOI:** 10.1098/rstb.2025.0196

**Published:** 2026-10-15

**Authors:** Matthew J. Hansen, Palina Bartashevich, Pia Bideau, Fritz A. Francisco, David James, Max Licht, Félicie Dhellemmes, Alicia L. Burns, Hector Villalobos-Ortiz, Sofia Ortega-Garcia, Felipe Galván-Magaña, Rogelio González-Armas, Fernando R. Elorriaga-Verplanken, Jens Krause

**Affiliations:** ^1^ Department of Fish Biology, Fisheries and Aquaculture, Leibniz-Institute of Freshwater Ecology and Inland Fisheries, Berlin, Germany; ^2^ Faculty of Life Sciences, Humboldt University of Berlin, Berlin, Germany; ^3^ Cluster of Excellence ‘Science of Intelligence’, Technical University of Berlin, Berlin, Germany; ^4^ Multidisciplinary Institute in Artificial Intelligence, Grenoble Alpes, Saint Martin d'Heres, France; ^5^ Department of Biology, Boston University, Boston, MA 02215, USA; ^6^ Max Planck Institute for Human Development, Berlin, Germany; ^7^ Instituto Politécnico Nacional, Centro Interdisciplinario de Ciencias Marinas (CICIMAR-IPN), La Paz, Baja California Sur, Mexico

**Keywords:** group-hunting, capture rates, predator–prey, body size, *Kajikia audax*, *Coryphaena hippurus*, manoeuvrability, nature of interactions, mutualism, competition

## Abstract

Terrestrial multi-species predator feeding aggregations (MPFAs) are characterized by contest competition, but scramble competition is likely more common at MPFAs in pelagic waters. However, empirical investigations are rare. Aerial video was used to estimate abundances of striped marlin and mahi-mahi alongside their attack and capture rates and spatio-temporal positioning as they competed at prey schools. Mahi-mahi had higher capture efficiencies than marlin, and the presence of marlin did not affect mahi-mahi predatory behaviour. Conversely, the presence of mahi-mahi in smaller abundances may benefit marlin. Marlin attack rates on the school were not affected by mahi-mahi presence, but net capture rates increased, likely explained by a shift in marlin hunting dynamics where marlin increased the proportion of their total captures that were individual fish isolated from the prey school. Estimated abundances of mahi-mahi, however, showed a negative relationship with marlin abundance. As mahi-mahi monopolized the space closest to the prey school, where they made rapid and efficient attacks on the prey school, in large abundances they may interfere with marlin hunting behaviour, causing them to abandon the hunt. The persistence of scramble or contest competition at MPFAs in pelagic waters may therefore be partially modulated by group size.

This article is part of the theme issue ‘Multi-predator feeding aggregations in pelagic waters’.

## 1. Introduction

Many ecologically important animal aggregations are characterized by the presence of multiple competing predator species (‘multi-species predator feeding aggregations’—MPFAs). These events provide great opportunities to assess how competitive dynamics are influenced by differences in morphology and attack behaviour among predators. Upon aggregating on a plentiful food resource, competition between animals can theoretically be categorized into one of two extremes: (i) pure contest competition, where one or a part of the competing group wins access to the resource (usually via physicality) and the other competitors lose out; and (ii) pure scramble competition, where individual access decreases proportionally to group size [1–3]. Most competitive interaction lies within these two extremes.

Central to the dynamics of competitive animal aggregations is the ability of one individual or a small group to monopolize the resource, and this competition for resource monopolization characterizes terrestrial MPFAs at carrion [4–7]. Between-species dominance hierarchies control prey access at carrion and are commonly determined by linear size/strength-relationships [8–15]. This dynamic involves competing species accessing prey sequentially, rather than simultaneously. These hierarchies, however, can be mediated by group size, which increases the likelihood that groups of subordinates can successfully defend or displace dominants [8,10,11,13,16–25]. As different species do not feed simultaneously, it is the absence of dominant species (even temporarily) that often provides the opportunity for subordinate species to feed [5,9,26] and commonly subordinates consume different and less nutritious tissue types [4,26–28].

The dynamics of terrestrial, contest-dominated competition are unlikely to seamlessly translate to other major MPFAs, such as those occurring in pelagic waters. In this environment, the most common resource for a wide variety of higher trophic level predators is not carrion but large groups of small, schooling invertebrates or fish, owing to an abundance of single-celled primary production and an overall low mean density of resources [29]. Since many predator species are specialized in exploiting the same type of locally abundant, heterogeneously distributed prey, and considering many of the predator species’ groups are composed of non-kin without an incentive to share or collectively defend the resource, there is potential for scramble competition. Critically, characteristics of prey schools make them difficult to monopolize.

One major characteristic making schooling prey difficult to monopolize is that they are inherently mobile and divisible, composed of bite-sized and relatively more manoeuvrable prey items that evade attacks as a collective [30–32]. Another characteristic is that the energetic investment required for resource defence increases with the number of competitors, and this effect is amplified in a three-dimensional, structureless environment, which allows for multiple points of prey access [33]. These factors make resource monopolization and contest competition unlikely at MPFAs in pelagic waters. Instead, scramble-competition may be predominant and competitive interactions likely lack dominance hierarchies [34,35]. However, even without overtly aggressive interactions ordering prey access, some animal species may outperform others within a scramble-competition context. For example, if some competitors achieve sufficiently high attack rates, other competitors’ intake rate may fall below a critical level, causing them to give up, effectively creating a short-term winner in the competition. To assess the dynamics of scramble competition at MPFAs, an estimation of predator number, alongside attack behaviour and capture rates, is required.

The intake rate of bottlenose dolphins, *Tursiops truncatus,* has been shown to decrease in the presence of silky sharks, *Carcharhinus falciformis* [35]; however, the intake rate of the sharks was not quantified. To date, the intake rates of two interacting heterospecific predators competing over the same prey school have only been quantified in California sea lions, *Zalophus californianus*, and striped marlin, *Kajikia audax*, in Baja California, Mexico. California sea lions interfered with striped marlin's ability to access prey schools, decreasing striped marlin attack and capture rate [36]. The dynamics of competition, however, may be specific to the combination of competing species. Considering the diversity of predator–predator interactions occurring at MPFAs in pelagic waters [37,38], overall, we know relatively little about what the presence and behaviour of heterospecific competitors have on the attack and capture rate of different species.

Mahi-mahi, *Coryphaena hippurus*, is another predator that has been observed alongside striped marlin at MPFAs, inviting investigation and comparison to the striped marlin–California sea lion interaction. Striped marlin and mahi-mahi are widely distributed, highly migratory teleosts that support both commercial and recreational fisheries [39,40]. They exemplify the energy-maximization lifestyle, likely wagering a high metabolic rate against high consumption rates [41,42], and have a range of behavioural, physiological and morphological traits associated with predatory life in the pelagic environment. They have fast growth rates and maturation times [43–45], recover quickly from exercise and have fast maximum and sustained speeds [46,47]. Striped marlin and mahi-mahi, therefore, share many traits and, compared with other common dyadic predator–predator interactions occurring at MPFAs in pelagic waters (e.g. cetaceans and birds), the two species could be considered phenotypically similar. However, while the two predators are both relatively large teleost species, striped marlin are significantly longer than mahi-mahi even when omitting their bill from calculations (by approx. 40%; striped marlin—125–180 eye-fork length [34,48,49]; mahi mahi—82–112 cm fork length [50]), and mahi-mahi are extremely laterally compressed [44,51,52]. Speed increases with body length up to a certain point, and manoeuvrability decreases with body length [53–56]. Thus, when capturing prey, mahi-mahi potentially rely more on manoeuvrability, and striped marlin on higher maximum speeds (or use of their bill) [52,57,58]. This is important in the context of competitive interactions at MPFAs as the effect of morphology and kinematics of predator attack behaviour may influence species’ respective tactics during a hunt [59,60].

Pelagic prey schools are a finite resource, inherently divisible and are attacked by a diversity of animals during MPFAs, promoting scramble competition conditions. It is only recently that we have the technology to measure the attack and capture rates and spatio-temporal positioning of predator and prey to explore the nature of competition under these ecological conditions. Moreover, an added curiosity is that because competitors at MPFAs are heterospecific, we can explore the dynamics of competition between competitors with potentially varying resource acquisition performance levels or tactics. In this study, we use aerial video from unmanned aerial vehicles (UAVs) to compare the hunting behaviour of mahi-mahi (* N* = 9 hunting sequences) and striped marlin (*N* = 13 hunting sequences) when alone and in the presence of each other (*N* = 10 hunting sequences). It is unclear which species (if any) dominates during competitive interactions given that striped marlin are larger and stronger and mahi-mahi are smaller and therefore more manoeuvrable. Differences in relative frequency may play a role because it has been shown that smaller predators, when in large numbers, can oust one or a few larger predators. Preliminary observations of large mahi-mahi schools (>50 individuals) with relatively few or no marlin present led us to predict a negative correlation between mahi-mahi and striped marlin abundances. We predict that when mahi-mahi are present in smaller numbers and hunting alongside striped marlin, the similarities between the two species will lead to interference competition as the two teleosts compete for prey access. We predict this interference competition will reduce the attack and capture rates of both predator species.

## 2. Methods

Aggregations of marine predators feeding on schools of prey fish were filmed from above through the water column using UAVs (DJI Phantom 4 Pro v. 2.0, 2.7/4k, 30/60 fps, circular polarized filter) in the Pacific Ocean offshore Baja California Sur, Mexico, in November 2021, 2022, 2023 and 2024 (see figure 1a and supplementary material for more details).

### (a) Predator abundances

Mahi-mahi were only sighted at aggregations in 2023 (not in 2021, 2022 or 2024). In 2023, there were 41 hunting sequences where environmental conditions allowed for estimations of the number of mahi-mahi and the number of striped marlin present during the hunt. For each of these 41 hunting sequences in 2023 (*N* = 41, mean ± s.e.m. length of 298 ± 45 seconds), at 30 second intervals, a still frame of the video was taken and imported to ImageJ to count the number of mahi-mahi and the number of striped marlin visible. California sea lions were also a common subsurface predator, so their numbers were counted as well. Visualization of predators was enhanced by playing the video for a few seconds.

### (b) Nature of interactions

Hunting sequences with California sea lions and other confounding predators were disregarded from future analyses (see supplementary material for details), which left *n* = 17 hunting sequences from 2023, where visibility was sufficient to record attacks and captures. These hunting sequences could be classified as either *only mahi* (*n* = 6; where mahi-mahi were the only subsurface predator present), *mixed-species* (*n* = 10; where both mahi-mahi and striped marlin were present) or *only marlin* (*n* = 1; where striped marlin were the only subsurface predator present). However, to increase replicate size for the *only mahi* predator composition treatment, an additional three *only mahi* hunting sequences from 2023 were included that also had California sea lions present (making a total of *n* = 9 *only mahi* hunting sequences). It was not possible to record captures in the three additional hunting sequences from 2023 with sea lions present. Only attacks were recorded. Relevant analyses were repeated both with and without the three additional hunting sequences that included sea lions, demonstrating that their inclusion did not change the validity of the results but only increased statistical power (see supplementary material for model outputs). Because there was only a single *only marlin* hunting sequence in 2023, the majority of *only marlin* hunting sequences used for analysis were collected from video recorded in 2021 (*n* = 5), 2022 (*n* = 4) and 2024 (*n* = 3), making a total of *n* = 13 *only marlin* hunting sequences. Thus, all hunting sequences used for analysis (*N* = 32, mean ± s.e.m. length of 380 ± 79 seconds) were classified as one of three mutually exclusive predator compositions: *only-marlin*, *mixed-species* and *only-mahi.*

Each hunting sequence was recorded as a continuous time series of predatory behavioural events using the software BORIS (v. 8.9.14) [61]. Behavioural events scored included *mahi-mahi prey school attack* and *striped marlin prey school attack* (and whether the attack was successful or not [binary variable: 0/1]);* mahi-mahi isolated prey capture* and *striped marlin isolated prey capture*;* estimated number of marlin* and *estimated number of mahi-mahi* (visible at each attack event) (see supplementary material for details).

The attack and capture frequencies for all the hunting sequences were investigated as time intervals between the respective events [36,62]. This was done for each species separately (*mahi attack interval* or *marlin attack interval* and *mahi capture interval* or *marlin capture interval*) as well as for the combination of predators (*combined attack interval* and *combined capture interval*). Calculations of capture intervals included captures of isolated prey items. However, we were also interested if the rate of isolation was affected by the predator compositions, so we also calculated the *combined isolation interval*. All interval data were multiplied by the estimated number of predators to provide per capita intervals. Any time interval that included time periods previously coded as visually obstructed or confounded by anthropogenic influence was excluded from analysis [36,62].

To assess how the prey school was divided between the two predator species, for each hunting sequence, the proportion of the *total captures* made by each species was calculated. Additionally, the proportion of each species’ *total captures* that were *isolate captures* or that resulted from attacking the prey school (*school captures* [*total captures*–*isolate captures*]) was also calculated. Finally, for each hunting sequence, the *capture efficiency* (likelihood of capture per attack of the prey school) was calculated. The capture efficiencies of the two predator species were calculated separately in the *mixed-species* predator composition (*mahi capture efficiency/ marlin capture efficiency*).

### (c) Spatial–temporal positioning

To assess how the positioning of predators may relate to their predator composition, we used Voronoi diagrams. A Voronoi diagram divides space into regions based on proximity [63]. For a set of points, each point is assigned its own region (a Voronoi polygon or cell) containing all locations closer to it than to any other point. Voronoi diagrams have been used in team sports, such as football and basketball, to quantify players’ control of the pitch by comparing the areas of their respective Voronoi cells [64–66]. We apply the same approach to animal behaviour, allowing us to quantify how individuals and species (mahi-mahi and striped marlin) share or dominate space during mixed-species hunting events. Throughout each hunting sequence, we annotated the head and tail of each predator as well as the circumference and direction of travel of the prey school. A Voronoi diagram was constructed over the annotations of predators and the prey school such that the latter also formed its own Voronoi cell. Here, a Voronoi cell is a region that contains all the space closest to its identifying predator/prey coordinates compared with any other predator/prey coordinate. Cells that shared an edge with the prey school cell were *first shell cells* and cells that did not share an edge with the prey school were *periphery cells. First shells* are further divided into *inner first shells* (completely bounded by other predator cells) and *outer first shells* (unbounded). Only frames where at least two individuals of each predator species were present were considered. We computed the normalized Voronoi area for each cell and also assessed the distribution of the proportion of predators occupying the *first shell*, the *periphery shell* and the *inner first* and *outer first shells* (seesupplementary material, figure S1 for details).

To assess the spatial–temporal dynamics of mahi-mahi and striped marlin attacks, we used still frames from the aerial video. All still frames were rescaled linearly to one flight height, i.e. we used 20 m as reference and rescaled everything to it. We used ImageJ to annotate the position (*x* and *y* coordinates in pixels) of a focal predator (mahi-mahi snout and striped marlin bill tip, *N* = 12 per species) and the closest prey fish, for 5 seconds prior to the attack and 5 seconds post-attack at half-second intervals. Pixels were converted to estimated distance in cm by using a prey fish (approx. 20 cm) for scale in each still frame.

We used recent advances in the interface of unmanned aerial vehicles and computer vision technology to collect geo-referenced tracked positions of mahi-mahi, striped marlin and the prey school from a 2-minute-long segment of a mixed-species hunt. Pixel positions of predators and prey were converted into geo-referenced world coordinates using the UAV's recorded GPS position and camera orientation [67]. We used the coordinate-based data from this 2-minute video to illustrate (i) the average distance to the prey school for each predator species and (ii) the between- and within-species inter-individual distances of the two predator species (see supplementary material for details).

### (d) Statistical analyses

The Bayesian hierarchical generalized models detailed below were built with the brm function of the brms library [68].

#### (i) Predator abundances

To assess the relationship between the abundance of mahi-mahi and California sea lions on striped marlin numbers, the response variable was the *estimated number of marlin* and the predictor variables were the *estimated number of mahi* and the *estimated number of California sea lions*. The regression family was set to zero-inflated negative binomial to handle the many occurrences where there were no striped marlin present. In the zero-inflation component, we included the *estimated number of mahi* as a predictor to test whether an increasing abundance of mahi-mahi significantly increased the likelihood of observing a zero striped marlin count (i.e. completely excluded striped marlin).

#### (ii) Influence of predator composition

To assess whether attack interval, capture interval and prey isolation intervals were affected by the predator composition (*marlin-only*/*mixed-species*/*mahi only*), we fitted each interval type as a response variable (*combined attack interval/combined capture interval/combined isolation interval*) and predator composition (*marlin-only*/*mixed-species*/*mahi only*) as a predictor variable. Hunting sequence ID was included as a random intercept (applies to all future models). The regression family was set to ‘Gamma (link = ‘log’)’.

#### (iii) Capture efficiencies

To assess whether there were differences in capture efficiencies between the two species under different predator compositions, we fitted species-specific *capture efficiency* as the response variable and predator composition (*marlin-only*/*marlin-mixed/mahi-mixed/mahi-only*) as the predictor variable (with *mixed species* split into two species-specific levels). A beta regression was used to model the continuous proportion data and to account for heteroscedasticity inherent in proportional outcomes. A single observation of 0 was adjusted using a standard transformation to slightly ‘shrink’ any exact 0s or 1s inward, ensuring all values fell within the (0,1) interval, while preserving the relative rank and distribution of the data [69]. Odds ratios and 95% highest posterior density (HPD) intervals were computed using pairwise contrasts based on the posterior distributions of the model.

#### (iv) Nature of interactions between striped marlin and mahi-mahi

*Attack and capture intervals*: To assess to what extent striped marlin attack behaviour was affected by the presence of mahi-mahi, we omitted mahi-only hunting sequences and fitted marlin attack interval/marlin capture interval (rather than combined attack interval/combined capture interval) as the response variable and predator composition (marlin-only/mixed-species) was set as the predictor variable. The regression family was set to ‘Gamma (link = ‘log’)’.

*Proportion of captures that were isolates.* To assess whether the presence of mahi-mahi affected the type of captures striped marlin were making (*isolated captures* versus *school captures*), the data were filtered to only include observations that were captures by striped marlin. *Isolated capture* (yes/no) was fitted as a binary response variable and predator composition (*marlin-only*/*mixed-species*) was fitted as the predictor variable. The regression family was set to ‘Bernoulli’.

*Mahi behaviour.* To test whether mahi-mahi behaviour was affected by the presence of striped marlin, the same three analyses (attack intervals, capture intervals and proportion of captures that were isolates) were run, but *marlin-only* hunting sequences were omitted rather than *mahi-only* hunting sequences.

*Transitional probabilities.* Finally, to further assess how the attack dynamics were impacted by species, within the *mixed-species* hunting sequences we calculated the attack transition probabilities [36]. We first calculated raw transition probabilities as the proportion of each attack type that was followed by either the same or the other predator species. To account for predator composition (relative estimated abundance of each predator species), we then calculated the expected probability that the next attacker would be striped marlin or mahi-mahi based on the relative numbers of each species present. Observed transitions were compared against these abundance-based expectations.

We formally tested whether the species identity of the previous attacker influenced the identity of the next attacker after accounting for predator composition using a binomial generalized linear model. The response variable was whether the next attack was by striped marlin, and the predictor was the identity of the current attack. To correct for predator composition, the logit-transformed expected probability of a striped marlin next attack, derived from relative group size, was included as an offset term. Thus, the model tested whether successive attacks were non-random with respect to species identity after controlling for the relative abundance of striped marlin and mahi-mahi.

#### (v) Resource partitioning

To assess how the shared resource was divided between the two species within the *mixed species* predator composition, we conducted a paired one-sample *t*-test comparing the observed proportion of *total captures* to the expected proportions—based on the mean estimated group size of the species observed within each *mixed species* hunting sequence. Following on from the earlier analysis assessing whether the presence of mahi-mahi affected the type of captures striped marlin were making, we conducted two further paired one-sample *t*-tests comparing the observed *isolated captures* and *school captures* to the expected proportions based on the mean estimated group size. Analyses were not conducted separately for each species owing to the complementary nature of the data (i.e. if one species was overrepresented, the other must be underrepresented). Results are reported for mahi-mahi, but the inverse results are inferred for striped marlin.

#### (vi) Spatial analyses

To assess for differences in normalized Voronoi area between the two species, the response variable was the *normalized Voronoi area* and the predictor variable was *species*. The regression family was set to Gamma with log link as the data are continuous and strictly positive and skewed.

To assess whether mahi-mahi and striped marlin differed in their attack dynamics, we tested whether their distances from the prey school were different over time (for 5 seconds prior to the attack and 5 seconds post-attack at half-second intervals). The response variable was *distance to the prey school* and the predictor variables were *species*, *time* and their interaction. *Time* was treated as a factor to allow nonlinear temporal patterns. Attack ID was fitted as a random intercept to account for repeated measures within individuals. The regression family was set to Gamma with log link. Posterior contrasts between species were computed at each time point using the emmeans package, with results presented as odds ratios of expected distances and associated 95% HPD intervals.

For the Bayesian hierarchical generalized models detailed above, default priors were used for all models. Four chains were run with 4000 iterations, of which the first 2000 iterations were discarded as warmup. Convergence of Markov chains was verified with visual inspection and the Gelman-Rubin statistic (Rhat). Rate ratios and 95% credible intervals (CI) were computed using pairwise contrasts based on the posterior distributions of the model. Credible effect differences in the posterior distributions were assessed by checking if the 95% CIs excluded zero (and/or if posterior median rate ratio CIs excluded one). CIs that include zero suggest that there is uncertainty in the effect.

## 3. Results

### (a) Predator abundances

Both the abundance of mahi-mahi (Est. −0.04, 95% CI [−0.06, −0.02]) and abundance of California sea lions (Est. −0.26, 95% CI [−0.49, −0.05]) are credibly associated with a lower abundance of striped marlin. The presence of sea lions had a stronger negative association than the presence of mahi-mahi; for each additional mahi-mahi, there was an approximately 5% decrease in striped marlin numbers (approx. 0.03 individuals) and for each additional California sea lion, an approximately 25% decrease in striped marlin (approx. 0.16 individuals). There was a positive relationship between the number of California sea lions and the number of mahi-mahi (supplementary material, figure S2).

Of the observations used in the model, 50% (233/463) had no striped marlin present. Less than 5% of the 230 observations with striped marlin present occurred when there were 20 or more mahi-mahi present (supplementary material, figure S2c). However, posterior distributions for the zero-inflated part of the model suggested the effect was uncertain. Therefore, there was not evidence that a particular degree of mahi-mahi abundance completely excluded marlin (estimated number of mahi-mahi—ZI part Est. 0.02, 95% CI [−0.11, 0.09]).

### (b) Influence of predator composition

Before assessing whether the presence of mahi-mahi or striped marlin affects the attack and capture interval of the other species, we assessed how attack and capture intervals (irrespective of predator species) were affected by the predator composition of the hunting group (*mahi only*,* marlin only* or *mixed species*; supplementary material, figures S3 and S4).

*Mahi-only* hunting sequences had a credible negative effect on attack intervals, with an approximately 60% reduction in attack interval time compared with *marlin-only* hunting sequences (*mahi-only* Est. −0.91, 95% CI [−1.28, −0.54]) (approx. 58.6 to approx. 23.8 seconds). *Mixed-species* hunting sequences also had a credible negative effect on attack intervals, with an approximately 48% reduction in attack interval time compared with *marlin-only* hunting sequences (Est. = −0.65, 95% CI [−1.02, −0.29]), corresponding to a decrease from approximately 58.6 to 30.6 seconds (supplementary material, figure S4a,b). Rate-ratio contrasts among predator-composition levels indicated that attack intervals were credibly shorter in both *mixed-species* and *mahi-only* hunting sequences than in *marlin-only* hunting sequences. Relative to *marlin-only* sequences, focal attack intervals were shorter in *mixed-species* sequences (RR = 0.52, 95% HPD interval [0.37, 0.78]) and in *mahi-only* sequences (RR = 0.40, 95% HPD interval [0.29, 0.61]). By contrast, focal attack intervals were not credibly different between *mixed-species* and *mahi-only* hunting sequences (RR = 0.78, 95% HPD interval [0.53, 1.20]).

Capture intervals of *mahi-only* and *mixed-species* hunting sequences (with captures by both species combined) were estimated to be credibly shorter than those of *marlin-only* hunting sequences. Relative to *marlin-only* sequences, capture intervals were approximately 66% shorter in *mahi-only* sequences (Est. = −1.09, 95% CI [−1.86, −0.30]), decreasing from about 135.6 to 45.9 seconds, and approximately 60% shorter in *mixed-species* sequences (Est. = −0.91, 95% CI [−1.52, −0.30]), decreasing from about 135.6 to 54.3 seconds (supplementary material, figure S4c,d). Rate-ratio contrasts among predator-composition levels indicated that capture intervals were credibly shorter in both *mixed-species* and *mahi-only* hunting sequences than in *marlin-only* hunting sequences. Relative to *marlin-only* sequences, capture intervals were shorter in *mixed-species* sequences (RR = 0.40, 95% HPD interval [0.24, 0.87]) and in *mahi-only* sequences (RR = 0.34, 95% HPD interval [0.17, 0.92]). By contrast, capture intervals were not credibly different between *mixed-species* and *mahi-only* hunting sequences (RR = 0.84, 95% HPD interval [0.41, 2.35]).

*Mixed-species* hunting sequences were associated with credibly shorter isolation intervals than *marlin-only* hunting sequences. Relative to *marlin-only* sequences, isolation intervals were approximately 67% shorter in *mixed-species* sequences (Est. = −1.10, 95% CI [−1.82, −0.36]), decreasing from about 233.0 to 77.0 seconds. Isolation intervals in *mahi-only* hunting sequences were also estimated to be shorter than in *marlin-only* hunting sequences, by approximately 60% (Est. = −0.92, 95% CI [−2.03, 0.15]), decreasing from about 233.0 to 94.2 seconds, but this effect was uncertain. Rate-ratio contrasts further illustrated this pattern: isolation intervals were credibly shorter in *mixed-species* than in *marlin-only* hunting sequences (RR = 0.33, 95% HPD interval [0.18, 0.87]), whereas there was no credible difference between *mahi-only* and *marlin-only* hunting sequences (RR = 0.40, 95% HPD interval [0.16, 1.93]) and no credible difference between *mixed-species* and *mahi-only* hunting sequences (RR = 0.83, 95% HPD interval [0.19, 2.26]). Overall, *mixed-species* hunting sequences have reduced attack, capture and isolation intervals compared to *marlin-only* hunting sequences. The *mixed-species* interval values are a combination of the activities of both predator species present in the same hunting sequence (i.e. mahi-mahi and striped marlin). Thus, the shorter intervals in the *mixed-species* hunting sequences are likely driven by the generally higher attack and capture rates of mahi-mahi compared to striped marlin.

### (c) Capture efficiencies

In general, mahi-mahi had higher capture efficiencies than striped marlin, regardless of predator composition (figure 1b) (approx. 30–44% for mahi-mahi compared with approx. 21% for striped marlin). We compared the *capture efficiency* within and between species across predator compositions using pairwise odds ratio contrasts between predator composition treatment group levels and 95% HPD intervals (supplementary material, figure S4). Mahi-mahi hunting in mixed-species groups (*mahi mixed*) had credibly higher capture efficiencies compared with striped marlin hunting alone (*marlin only* versus *mahi mixed* OR 0.33, 95% HPD [0.18, 0.52]) as well as striped marlin hunting in mixed-species groups (*marlin mixed* versus* mahi mixed* OR 0.22, 95% HPD [0.10, 0.38]). Additionally, mahi hunting alone (*mahi only*) had a credibly higher capture efficiency than striped marlin in mixed-species groups (*marlin mixed*) (*marlin mixed* versus *mahi only* OR 0.42, 95% HPD [0.16, 0.76]). Neither striped marlin nor mahi-mahi capture efficiencies when hunting alone were credibly different from when they were hunting in mixed-species groups (*marlin only* versus* marlin mixed* OR = 1.48, 95% HPD [0.74–2.68] or *mahi only* versus* mahi mixed* OR = 1.89, 95% HPD [0.93–3.28]) (figure 1; supplementary material, figure S5).

**Figure 1.**
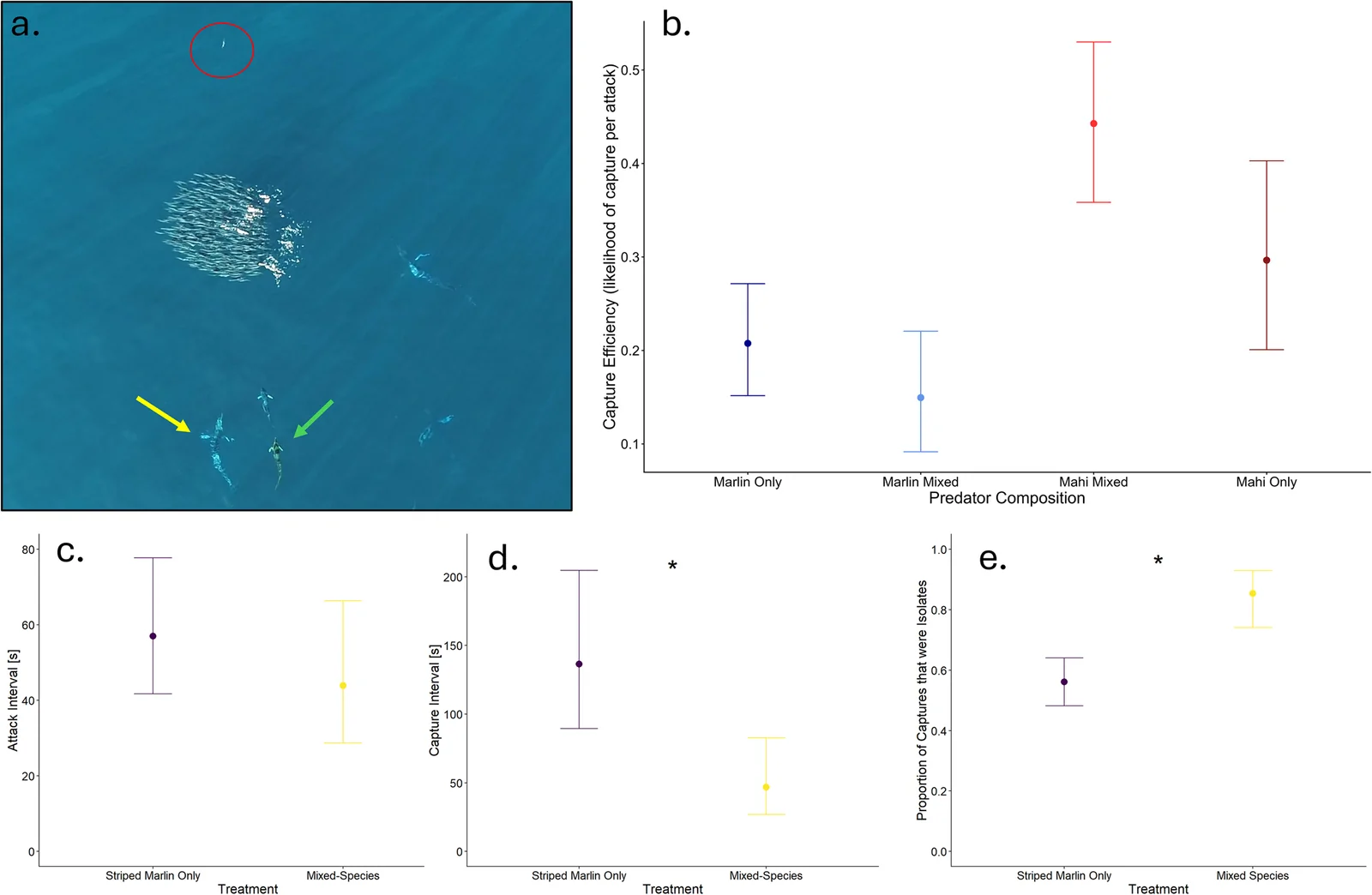
(a) Still frame from UAV showing striped marlin and mahi-mahi predating a prey school. An isolated sardine is circled in red; the yellow arrow points to a striped marlin, green arrow points to a mahi-mahi. (b) Capture efficiencies (likelihood of capture per attack on the school) for the two predator species (striped marlin and mahi-mahi) across the three predator compositions (marlin only/mixed-species/mahi only). (c) Attack intervals, (d) capture intervals and (e) proportion of captures that are isolates for striped marlin hunting alone (purple) and within mixed-species groups with mahi-mahi (yellow). Dots represent the median estimates from the posterior distribution and bars indicate 95% credible intervals.

### (d) Nature of interactions between striped marlin and mahi-mahi

The presence of mahi-mahi had no credible effect on the time interval between striped marlin attacks. Attack intervals were estimated to be approximately 23% shorter in *mixed species* hunting sequences than in *marlin-only* hunting sequences (Est. −0.26, 95% CI [−0.79, 0.25]), decreasing from about 57.0 to 43.9 seconds, but this effect was uncertain (figure 1c).

The presence of mahi-mahi had a credible effect on the time interval between striped marlin captures (Est. 1.07, 95% CI [−1.78, −0.36]; reducing from approx. 135.5 to 46.8 seconds; figure 1d) and a credible effect on the likelihood that striped marlin captures were on isolated prey (Est. 1.53, 95% CI [0.74, 2.38]; figure 1e). The probability of a capture being an isolated prey increases from 56% when striped marlin are hunting only with conspecifics, to 87% when hunting alongside mahi-mahi in mixed-species hunting sequences.

The presence of striped marlin had an uncertain effect on mahi-mahi attack interval (Est. −0.08, 95% CI [−0.41, 0.58]), capture interval (Est. 0.27, 95% CI [−0.53, 1.08]) and the likelihood that mahi-mahi captures were on isolated prey (Est. 0.46, 95% CI [−0.31, 1.20]; supplementary material, figure S6a–c).

Raw transition probabilities indicated that attack succession was strongly species-dependent. A *mahi-mahi attack* was more likely to be followed by another *mahi-mahi attack* (0.86) than a *marlin attack* (0.14). Striped marlin attacks were approximately as likely to be followed by another *marlin attack* (0.52) as a *mahi-mahi attack* (0.48). After correcting for predator group size, there was no evidence that *striped marlin attacks* were more likely to be followed by another *striped marlin attack* than expected by chance alone (*β* = −0.31, s.e.m. = 0.30, *z =* −1.03, *p* = 0.304). By contrast, following a *mahi-mahi attack*, the next attack was significantly more likely to be a mahi-mahi (*β* = –1.09, s.e.m. = 0.38, *z* = –2.88, *p* = 0.004), indicating stronger within-species clustering of *mahi-mahi attacks* after accounting for predator composition.

### (e) Resource partitioning

The proportion of *total captures* by mahi-mahi (mean ± s.e.m. = 0.65 ± 0.063) was not significantly different from the expected based on their estimated group size proportion (mean ± s.e.m. = 0.63 ± 0.069; *t* = 0.04, d.f. = 9, *p* = 0.96; (figure 2a). The proportion of *isolates* by mahi-mahi (mean ± s.e.m. = 0.45 ± 0.08) was significantly lower from the expected based on their estimated group size proportion (*t* = −2.32, d.f. = 9, *p* = 0.046; figure 2b), and the proportion of *school* captures (mean ± s.e.m. = 0.86 ± 0.04) was significantly higher than the expected based on their estimated group size proportion (*t* = 4.89, d.f. = 9, *p* < 0.001; figure 2c).

**Figure 2.**
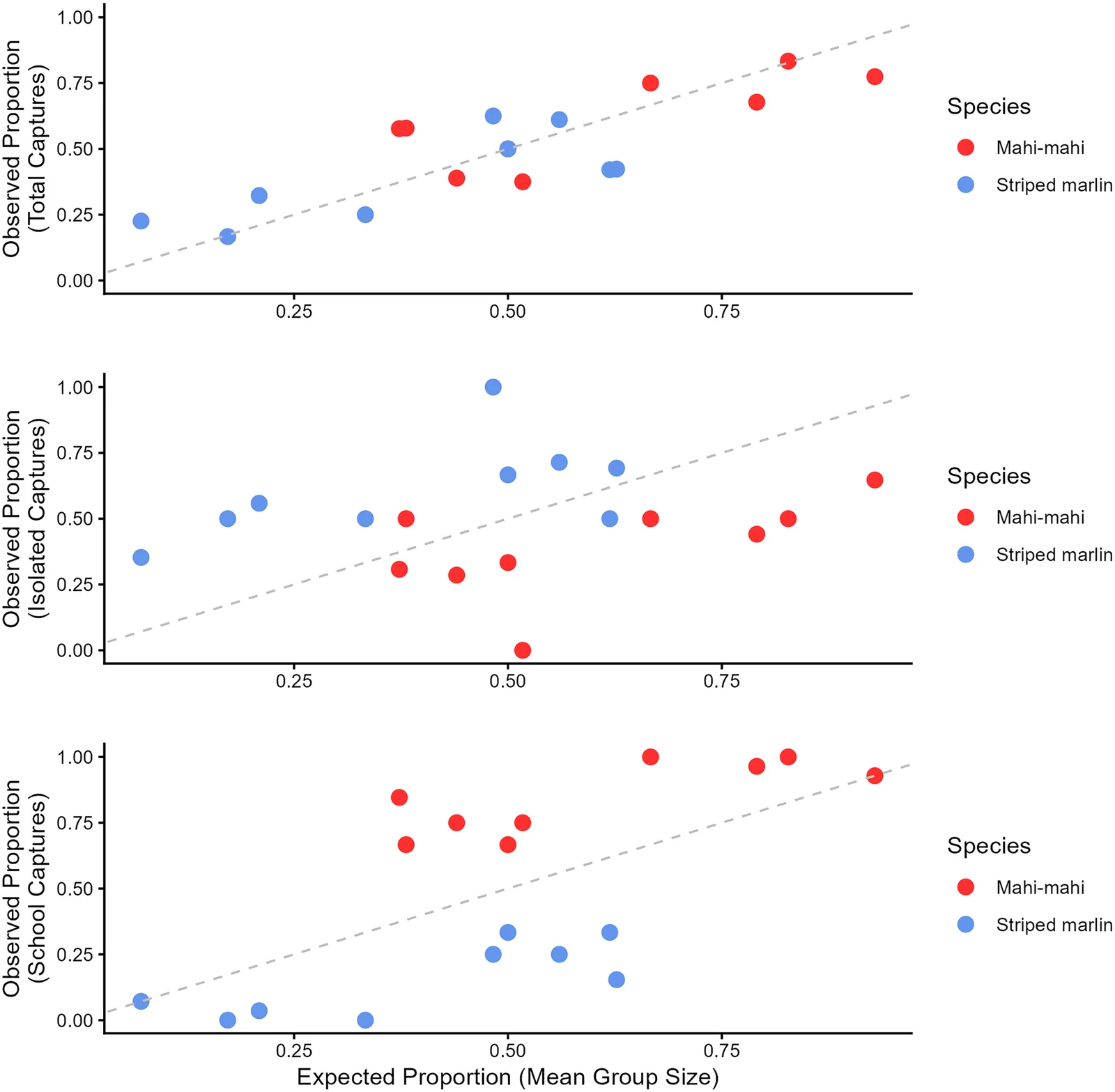
The observed (*y*-axis) and expected (*x*-axis) proportion of (a) total captures, (b) isolated captures and (c) school captures for mahi-mahi (red dots) and striped marlin (blue dots). Expected proportions are based on the estimated mean group size for each predator species within the mixed-species groups. Dotted lines represent where the observed proportion is exactly equal to the expected proportion. Points above the line are representative of observed proportions higher than expected based on estimated mean group size, and points below the line are representative of observed proportions that are lower than expected.

### (f) Spatial analyses

#### (i) Voronoi diagrams

Mahi-mahi and striped marlin were equally likely to be in the *first shell* (figure 3a,b) but mahi-mahi dominated the *inner first cells* and striped marlin the *outer first shells* (figure 3a,b). Striped marlin were more likely to be in *peripheral shells* than mahi-mahi (figure 3a,b). The ratio of samples where a mahi-mahi was in the same Voronoi cell as the prey school was 0.65, while for striped marlin it was 0.35. Therefore, in the majority of cases, mahi-mahi were closer to the prey school than striped marlin.

**Figure 3.**
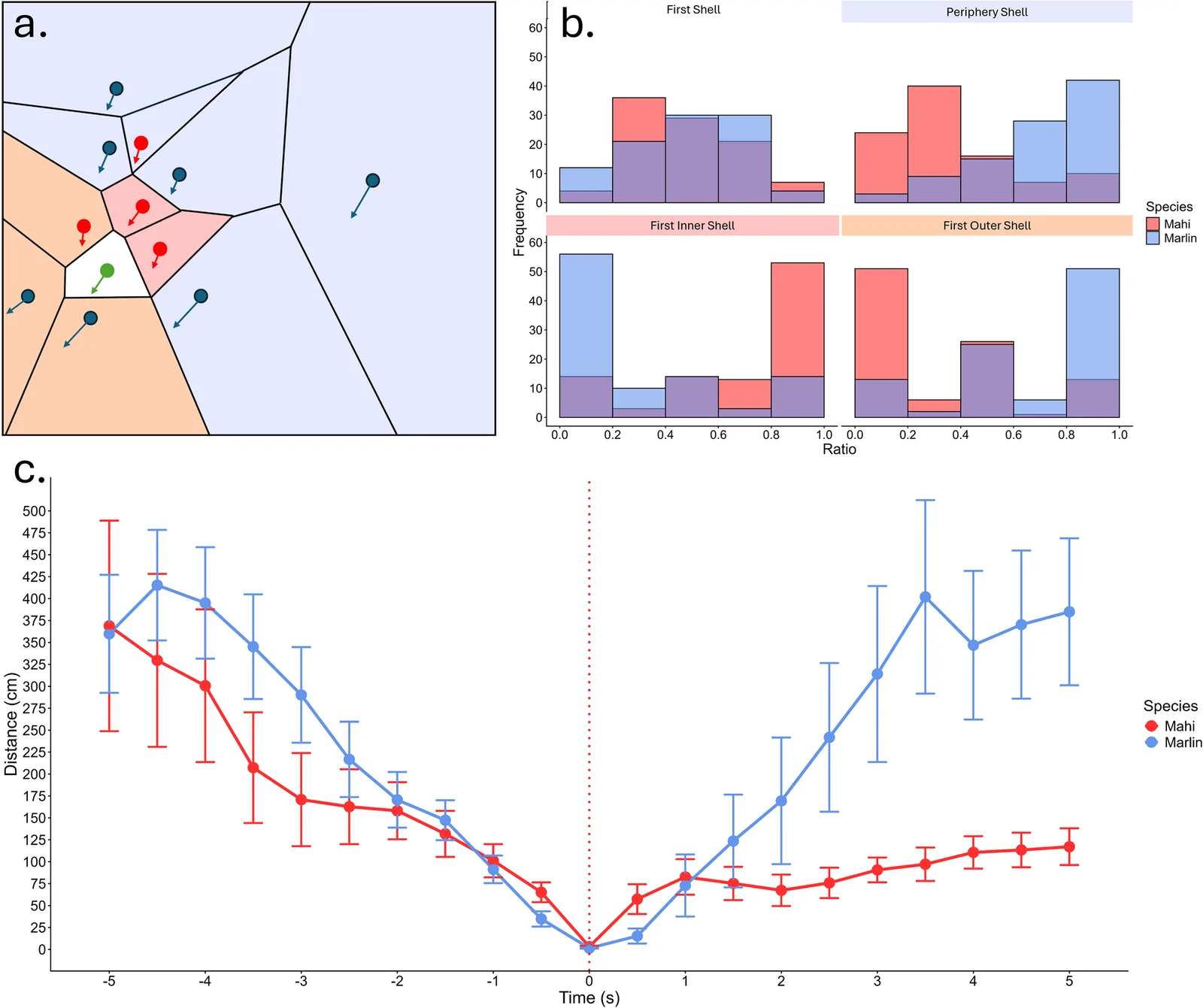
(a) Example of a Voronoi diagram of mahi-mahi (red dots) and striped marlin (blue dots) hunting a prey school (green dot). Arrows show direction of motion. Voronoi cells are delineated by black lines. For any of the dots, its Voronoi cell is the region of all locations closer to itself than any other dot. Red shaded cells represent inner first shell cells, orange shaded cells represent outer first shell cells and blue shaded cells represent periphery cells. First shell cells are all cells that share an edge with the prey school. Inner first shell cells are completely bounded, i.e. it has a neighbour on all sides. Outer first shell cells are unbounded. Periphery cells do not share an edge with the prey school. (b) Species positioning on Voronoi diagram: the distribution of the proportion of predators occupying the first shell around the prey school, the distribution of the proportion of predators occupying the periphery shells around the prey school, the distribution of the proportion of predators occupying the first inner shell around the prey school, and the distribution of the proportion of predators occupying the first outer shell around the prey school. (c) Distance to the nearest prey fish (cm) for 5 seconds pre and 5 seconds post mahi-mahi (red) and striped marlin (blue) attacks. Red dotted line is prey school contact point.

Striped marlin had significantly larger normalized Voronoi areas than mahi-mahi (*mahi only* Est. −0.74, 95% CI [0.58, −1.87]), approximately 1.73 times larger on average (supplementary material, figure S7).

#### (ii) Attack dynamics

There was no credible evidence for a difference in the distance of mahi-mahi or striped marlin to the prey school pre-contact (e.g. at −5 s: OR = 0.90, 95% HPD [0.17, 2.34]; at −0.5 s: OR = 2.37, 95% HPD [0.35, 6.21]. From 0.5 to 2.5 seconds, striped marlin tended to move further away than mahi-mahi, but the effect was uncertain (e.g. at 2.5 s: OR = 0.556, 95% HPD [0.13, 1.50]). From 3.5 seconds onward, however, there was credible evidence that striped marlin were further from the prey school than mahi-mahi (e.g. at 3.5 s: OR = 0.32, 95% HPD [0.07, 0.85]; and at 5 s: OR = 0.30, 95% HPD [0.06, 0.78]; supplementary material, figure S8). Striped marlin (approx. 3.8 m) were, on average, approximately three times farther away from the prey school than mahi-mahi (approx. 1.3 m) by the end of the 10-second observation window (figure 3c).

## 4. Discussion

Contrary to our prediction, there was no direct evidence for interference competition between the two teleost predators when they formed mixed-species hunting groups. Neither the attack rates of mahi-mahi nor marlin were affected by the presence of the other predator species. However, there was a negative relationship between the estimated abundances of mahi-mahi and striped marlin. Unexpectedly, although striped marlin showed no difference in attack rate on the prey school during mixed-species hunts, their capture rate was higher during mixed-species hunts. This pattern was not observed for mahi-mahi. This discrepancy might be explained by the way that striped marlin shifted their captures to isolated prey fish during mixed-species hunts, whereas mahi-mahi continued to capture the majority of their prey from attacks on the school. There is mounting evidence that lunge-feeding rorqual whales may benefit from the prey herding behaviour of other predators at MPFAs in pelagic waters ([70], see Krylova et al. [71]), and that aerial predators commonly rely on subsurface predators to drive prey to the surface and scatter prey [72], see Licht et al. [73]). However, there is little empirical data documenting an alteration to a species’ attack behaviour that enables them to maintain or increase capture rates in the presence of competing species (but see [74]). Estimations of how the shared resource was distributed between mahi-mahi and striped marlin within mixed-species groups show that, while both species captured prey in proportion to their relative numbers (supporting their temporary co-presence at the aggregation), mahi-mahi captured disproportionately more from direct attacks on the prey school and striped marlin disproportionately more isolated prey.

There was evidence of a higher rate of prey isolation in the mixed-species hunts compared with the marlin-only hunts, and while isolated and more vulnerable prey were conceivably equally available to both species of predator, spatial analyses show that marlin may have been best positioned to capitalize on these isolated prey fish. In spatial analyses, larger Voronoi areas are interpreted as dominance or control of the available space [65,66]. Striped marlin had larger Voronoi areas in general, compared with mahi-mahi, i.e. they were further from their nearest neighbours with more exclusive space around them. However, striped marlin only dominated the periphery of the hunting group. Striped marlin positioning and space use seem to have favoured the targeting of isolated prey fish rather than accessing the prey school within mixed-species hunting groups. This pattern potentially reflects their larger overall body size and lower manoeuvrability. Striped marlin are less manoeuvrable than mahi-mahi, with more limited dorso-lateral flexibility. They potentially require more space to position themselves before and after attacks, and perhaps fewer obstacles near the prey school to make their high-speed, straight-line attacks [57]. Conversely, mahi-mahi were more likely to be enclosed by other predators and closer to the prey school. In most samples (0.65), mahi-mahi were closer to the prey school than striped marlin (figure 4a,b). Assessments of the positioning of the different predators pre- and post- attacks also showed that, on average, mahi-mahi remained closer to the prey school after making an attack. Additionally, the transitional probabilities showed that mahi-mahi attacks were more likely to be followed by another mahi-mahi attack, even after accounting for the abundance of each predator species. This species attack clustering did not occur for striped marlin. Considering mahi-mahi had smaller inter-individual distances (figure 4c), were generally found closer to the prey school, had higher attack rates and showed evidence of species attack clustering, it is understandable how, in high abundances, they might swamp the resource, causing marlin to abandon the MPFA and search for a prey school with less competition. Mahi-mahi were only present in 2023, the year with the highest sea-surface temperature, suggesting the between-predator interactions observed in 2023 could occur again if mahi-mahi shift their range northwards in response to warm El Niño–Southern Oscillation (ENSO) years to a greater degree than striped marlin [75,76]. However, even with respect to ENSO, the two species have been found to occupy different trophic spaces [77], and both predator species are generalists. In the absence of data representing either species’ daily caloric intake or reproductive output, flow-on effects of interference competition to a negative effect at the population level remain uncertain.

**Figure 4.**
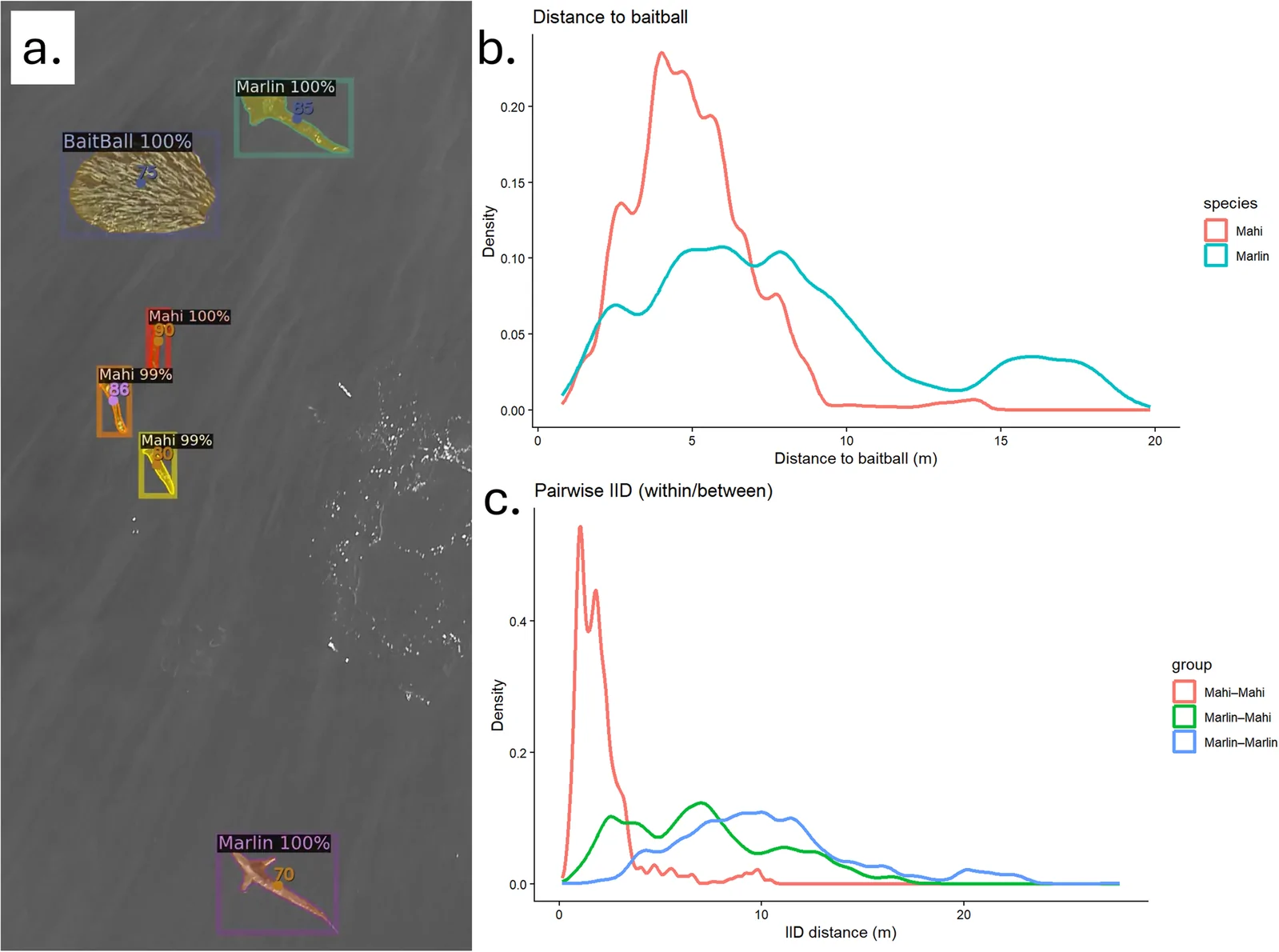
(a) Still frame output from tracking pipeline (supplementary material) after inference was performed on aerial video, showing detection accuracy (%) and object classification (mahi-mahi, striped marlin and prey school). Density curves represent kernel density estimates of frame-level distance measurements: distance to prey school (b), pairwise interindividual distances (c), calculated from the geo-referenced trajectories of all tracked individuals (mahi-mahi, red; striped marlin, blue; mixed, green) across the 2-minute video segment.

Predator body size and its effect on prey access and intake rates is likely an important factor determining the nature of interactions between predators at MPFAs (see Domenici et al. [78]). Rorqual whales likely benefit from prey being condensed by other predators and are thought to terminate mixed-species hunts by consuming large proportions of the available resource [70]. Descriptions of different dolphin species suggest that the larger species can temporarily dominate prey access to the school and cause other predators to scatter prior to attacks [79]. However, the attacks of silky sharks (the smaller predator) have been found to impede prey access by bottlenose dolphins (the larger predator) [35] (as well as striped marlin; M. J. Hansen, personal observation). And, here, results show that mahi-mahi (the smaller competitor) maintained their relatively higher attack rate and capture efficiency in the presence of a larger competitor (striped marlin), and got most of their captures from targeting the prey school. Sharks are more manoeuvrable than bottlenose dolphins, and mahi-mahi are more manoeuvrable than striped marlin, suggesting that manoeuvrability (which tends to be greater in smaller-bodied animals) may be an important and underappreciated characteristic determining competitive interactions between heterospecific predators at MPFAs. Striped marlin were able to increase their capture rate in the presence of the smaller-bodied mahi-mahi, but not by dominating prey school access. Rather, they exploited the increased amount of isolated prey that occurred during mixed-species hunts. Despite neither striped marlin nor mahi-mahi having a lower attack or capture rate in mixed-species groups, there was a negative relationship between mahi-mahi abundance and striped marlin abundance, with striped marlin being very rare when mahi-mahi abundance was greater than approximately 20 individuals. However, there was no strong evidence that a particular threshold number of mahi-mahi precluded marlin from the MPFA. This result was surprising considering only 5% of the observations with striped marlin present occurred when there were more than 20 mahi-mahi. However, there was a general positive association between mahi-mahi and California sea lion abundances, and even low numbers of mahi-mahi (i.e. <20) were associated with California sea lions 20% of the time. Considering the relatively stronger negative effect that California sea lions have on striped marlin presence compared with mahi-mahi, it is possible that striped marlin were excluded from aggregations with low numbers of mahi-mahi in part because of the additive effect of California sea lion presence. This pattern would have affected the model assessing whether a threshold degree of mahi-mahi abundance completely excluded marlin, as even low numbers of mahi-mahi had an exclusionary effect (potentially via the added influence of California sea lions).

Mahi-mahi attacks on the prey school were efficient in capturing prey. When attacking the prey school within mixed-species hunting groups, mahi-mahi capture efficiency (Mean ± s.e.m. = 0.45 ± 0.06, min = 0.16, max = 0.75) was approximately 3 times greater compared with striped marlin (Mean ± s.e.m. = 0.14 ± 0.03, min = 0.0, max = 0.25). Although laboratory research suggests the species is physiologically attuned to marrying a high activity rate with a high consumption rate [41], detailed information on their attack behaviour and capture rates from the wild was previously missing. Future work should now focus on more fine-scale analysis of both predator species’ attack kinematics and that of the prey to test what properties are responsible for mahi-mahi's high capture efficiencies. For example, perhaps mahi-mahi get closer to prey before prey initiates a late, sharp minute turn (‘turning gambit’ [80]). Or perhaps a relatively high turning radius to speed ratio allows mahi-mahi to compensate for the manoeuvrability of their prey [52,54]. These questions can potentially be tested by using the emerging technology illustrated in figure 4, where aerial videos of wild predator–prey interactions in pelagic surface waters can be converted into geo-referenced coordinate data.

In conclusion, whether the competitive interactions between striped marlin and mahi-mahi more closely resemble scramble or contest competition (which dominates terrestrial MPFAs) may depend on predator group size. When hunting alongside each other in mixed-species hunts, neither species’ attack rates on the prey school were negatively affected by the presence of the other species. However, while mahi-mahi maintained their high capture efficiencies via attacks on the prey school, striped marlin actually increased their capture rate in the presence of mahi-mahi by capturing a disproportionate amount of the isolated prey made available during mixed-species hunts. These dynamics of resource division allowed both predator species to capture prey in proportion to their relative abundance. This pattern may have emerged from differences in the predators’ morphology and attack kinematics, which potentially could also help explain the overall negative relationship between mahi-mahi and striped marlin abundances. Mahi-mahi are smaller, more manoeuvrable, have higher attack rates and stayed closer to the prey school post-attack. During mixed-species hunting sequences, striped marlin dominated the space further from the prey school and had relatively larger inter-individual distances, which potentially aided their capture of isolates, but could be detrimental to overall attack rate and captures when mahi-mahi abundances become very large. These results lead us to predict that both predator manoeuvrability and predator group size are important factors determining heterospecific differences in prey school access at MPFAs in pelagic waters. Under certain predator compositions, the less manoeuvrable predators at MPFAs may require shifts in their attack strategies to maintain capture rates.

## Supplementary Material



## Data Availability

Data are available as supplementary material. Supplementary material is available online [81].

## Theme

One contribution of 14 to a theme issue ‘Multi predator feeding aggregations in pelagic waters’.
