# Supplementary material for: The presence of mahi-mahi at multi-predator feeding aggregations modifies striped marlin attack behaviour
Source: Philos Trans R Soc Lond B Biol Sci. 2026 Oct 15;381(1961):20250196. doi: 10.1098/rstb.2025.0196 (PMC13670072; doi:10.1098/rstb.2025.0196)
Supplement: Supplementary file 1 [file rstb.2025.0196.zip › rstb.2025.0196/rstb20250196_si_001.docx]

**Supplementary Materials**

**Interference competition with mahi-mahi modifies striped marlin attack behaviour in multi-predator feeding aggregations**

**Hansen, M.J., Bartashevich, P., Licht, M., Dhellemmes, F., Burns, A., Villalobos-Ortiz, H., Ortega-Garcia, S., Galvan-Magana, F., Gonzalez-Armas, R., Elorriaga-Verplancken, F.R., Krause, J.**

**Methods**

Aggregations were located by visually scanning the horizon for seabirds from a 28ft Boston Whaler boat. Upon confirming the presence of subsurface predators, researchers launched an Unmanned Aerial Vehicle (UAV, DJI Phantom 4 Pro v.2.0, 2.7/4k, 30/60 fps, circular polarised filter) to record the predator-prey interactions from an altitude of 10-30m. Recording the subsurface predators with UAVs has no effect on their hunting behaviour^1^. Recording began once the prey school could be visualised on the screen. Aggregations were recorded continuously until batteries ran out or predator-prey interactions could not be visualised ^1^.

**Nature of interactions**

To assess how the predator composition affected attack and capture behaviour of striped marlin and mahi-mahi, hunting sequences that had sub-surface predators other than these focal species (confounding predators), for example, Californian sea lions, *Zalophus californianus* ^2^, were excluded from further analysis. This was because the presence and activity of these predators are a confounding factor, and it is known that sealions negatively affect the attack and capture rates of striped marlin through interference competition^2^. As all aggregations had aerial predation; it was not possible to filter out hunting sequences with sea-birds present.

The predatory behaviour of mahi-mahi and/ or striped marlin were coded using the software BORIS (v.8.9.14) ^3^. Video was replayed at 0.2x original speed and every mahi-mahi and striped marlin attack on the prey school was time-stamped (*mahi-mahi attack* / *striped marlin attack*). The timing of an attack was defined as the point in time when either the bill of the striped marlin or the snout of the mahi-mahi entered the prey school. If this point was not clearly identified because, for example, the predator attacked from below the prey school, or there were some obfuscating splashes, attack time was estimated either via an increase in tailbeat frequency or from prey escape behaviour ^1^. It was also noted during this process which sections of the time series were visually obstructed or confounded by the presence of anthropomophic influence (for example if the prey school temporarily moved outside of the video frame, if it dove too deep to visualise, or if boat wash or tourists were temporarily in frame). In addition to attack timing, it was coded whether the attack was successful or not (binary variable: 0/1). Successful attacks were those in which a prey fish was ingested by the attacking predator during the attack on the prey school or as a direct consequence shortly afterwards. The accuracy of assessing capture success from aerial video has previously been assessed for striped marlin by comparing it to syncronised underwater video ^1^. While this was not possible with mahi-mahi due to an absence of sufficient syncronised underwater video for this species, the captures of mahi-mahi are clearer than those of striped marlin due to their smaller relative size difference to the prey. Their ingestions are more exagerated than with striped marlin and are easily identified from aerial video. Aside from directly attacking prey schools, predators also captured prey fish that had become isolated from the school. These isolate captures are made by individuals that are not in the process of directly attacking the prey school ^1^. Therefore the captures of isolated prey items were coded as separate events for each predator species (*mahi-mahi isolate* / *striped marlin isolate*) within the ongoing time series of predator attacks. For each predatory event the number of striped marlin (*estimated number of marlin*) and number of mahi-mahi (*estimated number of mahi*) visible within the frame of the video were counted.

**Spatial analysis**

Supp Fig 1. Example of Voronoi diagram of mahi mahi (red dots) and striped marlin (blue dots) hunting a prey school (green dot). Arrows show direction of motion. Voronoi cells are delineated by black lines. For any of the dots, its Voronoi cell is the region of all locations closer to itself than any other dot. Red shaded cells represent inner first shell cells, orange shaded cells represent outer first shell cells and blue shaded cells represent periphery cells. First shell cells are all cells that share an edge with the prey school. Inner first shell cells are completely bounded, i.e. it has neighbour on all sides. Outer first shell cells are unbounded. Periphery cells do not share an edge with the prey school.

Voronoi area: Voronoi diagrams (including the area of Voronoi cells) have been used in sports analysis to indicate which team controls the most space and has an advantageous position in the game (Kim et al. 2004; Raabe et al. 2024; Fonseca et al. 2012). To account for different number of predators in each of our samples and the induced density effects, we compute the normalised Voronoi area as the Voronoi area each predator would get if the total space were split equally among all predators in the current sample. Namely, we compute the total area of the Voronoi bounding box (the same for all frames, $A_{bbox}$) and compute the relative area of the Voronoi cell with a predator $V_{i}$ to $A_{bbox}$ multiplied by the total number of predators in this frame, i.e., $\frac{A_{V_{i}}}{A_{bbox}}*N$. This way, if the normalised area equals 1 means the exactly fair share of the space by the predator, if the normalised area < 1 – the predator controls less area than the average predator would if space were split evenly, and if the normalised area > 1 – more space than fair share. Since the peripheral cells of Voronoi diagram occupy larger Voronoi areas by definition, we performed the analysis where we exclude the Voronoi cells on the periphery.

The distribution of the proportion of predators occupying the *first shell* around the prey school - for each frame, we counted the total number of *first shell* cells and calculated the ratio occupied by marlin (or mahi-mahi) relative to the total number of *first shell* cells.

The distribution of the proportion of predators occupying the *periphery shells* around the prey school - for each frame, we counted the total number of marlin (or mahi-mahi) and computed the ratio of marlin (or mahi-mahi) in the *periphery cells* to the total number of available marlin (mahi-mahi) in that frame.

The distribution of the proportion of predators occupying the *first inner shell* around the prey school - for each frame, we counted the number of *inner first shell* cells and computed the ratio occupied by marlins (or mahi-mahi) to this number).

The distribution of the proportion of predators occupying the *first outer shell* around the prey school - for each frame, we counted the number of *outer first shell* cells and computed the ratio occupied by marlins (or mahi-mahi) to this number.

Extraction of predator trajectories from UAV video: To quantify the spatial relationships between predators and prey school during mixed-species hunting events, we used a computer-vision workflow to extract geo-referenced trajectories of predators and prey from aerial video. Recent advances in the integration of unmanned aerial vehicles (UAVs) with automated object detection methods allow accurate tracking of animals in complex marine environments (Bideau et al. 2026).

The aerial video was paired with its corresponding drone flight log data. A convolutional neural network–based object detection framework was trained to identify striped marlin (*Kajikia audax*), mahi-mahi (*Coryphaena hippurus*), and the prey school. Training began from a pretrained object detection model provided in the Detectron2 model zoo ("COCO-InstanceSegmentation/mask_rcnn_X_101_32x8d_FPN_3x.yaml") and was subsequently fine-tuned using a custom dataset of 74 manually annotated frames (1,382 labelled objects) in COCO (Common Objects in Context) format. Model training was performed for 100,000 iterations on a local workstation equipped with an AMD Radeon 880M GPU.

Following training, the model was used to perform inference on video footage of a single 2-minute-long continuous hunting event. During inference, the model detected and classified animals (mahi-mahi, striped marlin, prey school) in each frame and estimated their positions within the image. Detected objects were then linked across consecutive frames using a distance-based tracking algorithm that assigns a consistent identity to detections occurring within a specified spatial threshold between frames. This procedure produced frame-by-frame trajectories for each detected predator and the prey school.

Labels assigned to each trajectory were then manually reviewed to ensure accuracy. Erroneous trajectories (e.g. glare mistaken for a predator, splashes mistaken for a prey school) were discarded. Pixel coordinates that formed the trajectories were then converted into geo-referenced world coordinates using the UAV’s recorded GPS position and camera orientation (see Bideau et al. 2026 for details of methodology). The resulting dataset contained time-resolved x,y positions for each trajectory expressed in meters relative to a geographic reference point. The notebooks used for model training, inference, trajectory extraction, and coordinate conversion are available for review upon reasonable request.

Calculation and visualisation of spatial distance distributions: To visualize spatial relationships between predators and the prey school, we calculated two frame-level spatial metrics from the geo-referenced trajectory data: (i) the distance between each predator and the centroid of the prey school and (ii) inter-individual distances among predators. Distances were calculated for every tracked individual at every video frame using distance between the corresponding x,y coordinates.

For predator–prey distance, the position of each predator in a given frame was compared with the centroid of the prey school in the same frame. For inter-individual distances, pairwise distances were calculated between predator positions within each frame. Distances were then grouped according to species identity to allow comparisons within and between predator species.

The distributions shown in Fig 4 represent kernel density estimates of these frame-level distances. All individual distance measurements across the analysed video segment were pooled by species and used to estimate the probability density of distances using a Gaussian kernel implemented in the geom_density() function in R (ggplot2 package).

**Results**

Supp Fig 2 (a) The relationship between the estimated number of striped marlin (y-axis) and estimated number of mahi-mahi (x=axis) observed during multi-predator feeding aggregations. (b) The relationship between the estimated number of striped marlin (y-axis) and estimated number of California sea lions (x=axis) observed during multi-predator feeding aggregations. (c) The cumulative percentage of observations with at least one striped marlin present (y-axis) in relation to the estimated number of mahi-mahi (x-axis). Green dotted line represents 95%. (d) The relationship between the estimated number of mahi-mahi (y-axis) and estimated number of California sea lions (x=axis) observed during multi-predator feeding aggregations.

Supp Fig 3 The relationship between the estimated number of predators in each treatment (regardless of species) and predicted time between attacks in seconds. Here, predicted time between attacks is at the level of the group. *Marlin only* treatment (black line) shows a flatter decrease with additional predators compared to the treatments with mahi-mahi present. In the *mixed species* (blue line) and *mahi only* (red line) treatments there is a stronger exponential effect of additional predators.

Supp Fig. 4 Raw data of (a) attack (c) capture and (e) isolation interval (seconds, y-axis) for different predator composition treatments (marlin only, mixed-species and mahi-mahi) (x-axis). Orange boxplots and dots represent striped marlin and blue boxplots and dots represent mahi-mahi data. Conditional effects of (b) attack, (d) capture and (f) isolation intervals (seconds, y-axis) for different predator composition treatments (marlin only, mixed-species and mahi only). Centre dots represent the median estimates from the posterior distribution and bars indicate 95% credible intervals.

Supp Fig 5 Forest Plot of Treatment Contrasts (Odds Ratio) (x-axis) for differences in capture efficiencies between species (striped marlin and mahi-mahi) within different predator compositions (y-axis). For each pairwise contrast the point shown is the posterior median of the odds ratio distribution and the bars represent the 95% Highest Posterior Density (HPD) intervals. An OR >1 suggests higher odds in the numerator group, and OR<1 suggests lower odds in the numerator group and an OR= 1 mean no difference. If the 95% HPD interval includes one therefore that means there is a 95% probability that the true odds ratio lies with the no effect range (OR=1).

Supp Fig. 6 (a) attack, (b) capture and (c) proportion of captures that are isolates for mahi-mahi hunting alone (purple) and within mixed species groups with striped marlin (yellow). Dots represent the median estimates from the posterior distribution and bars indicate 95% credible intervals.

Supp Fig 7 Normalised Voronoi area (y-axis) for each predator composition treatment (marlin only, mixed species, mahi only) (x-axis) coloured by species (red is mahi-mahi and blue is striped marlin). Heavy line within boxplot indicates median, boxes span from 25 to 75 percentile (the interquartile range; IQR), and whiskers extend 1.5*IQR.

Supp Fig 8 Posterior contrasts of distance from prey school between mahi-mahi and striped marlin (Distance Ratio, y-axis) for 5 sec pre and 5 sec post predator attacks (x-axis). Time 0 is point of prey school contact. Ratios from Gamma GLMM (brms); points are posterior medians ± 95% HPD, black dots represent uncertain differences between predators and red dots represent credible differences between distances.

*Statistical modelling*

**Predator abundances**

Effect of mahi-mahi and sealion abundance on striped marlin abundance:

Family: zero_inflated_negbinomial

Links: mu = log; shape = identity; zi = logit

Formula: Num_Marlin ~ Num_Mahi + Num_Sealion + (1 | ID)

zi ~ Num_Mahi

Data: data_predabund (Number of observations: 463)

Draws: 4 chains, each with iter = 4000; warmup = 2000; thin = 1;

total post-warmup draws = 8000

Multilevel Hyperparameters:

~ID (Number of levels: 40)

Estimate Est.Error l-95% CI u-95% CI Rhat Bulk_ESS Tail_ESS

sd(Intercept)	2.10	0.42	1.45	3.09 1.00	1746	2899

Regression Coefficients:

Estimate Est.Error l-95% CI u-95% CI Rhat Bulk_ESS Tail_ESS

Intercept	-0.45	0.49	-1.53	0.39 1.00	1268	2455

zi_Intercept	-6.31	1.53	-10.17	-4.13 1.00	8193	3615

Num_Mahi	-0.04	0.01	-0.06	-0.02 1.00	4902	5703

Num_Sealion	-0.26	0.11	-0.49	-0.05 1.00	4833	5130

zi_Num_Mahi	0.02	0.05	-0.11	0.10 1.00	4627	2877

**Influence of predator composition**

Effect of predator composition on attack interval:

Family: gamma

Links: mu = log

Formula: TimebwAttacks_focal ~ Treatment + (1 | ID)

Data: data (Number of observations: 2443)

Draws: 4 chains, each with iter = 4000; warmup = 2000; thin = 1;

total post-warmup draws = 8000

Multilevel Hyperparameters:

~ID (Number of levels: 32)

Estimate Est.Error l-95% CI u-95% CI Rhat Bulk_ESS Tail_ESS

sd(Intercept	0.42	0.07	0.31	0.57 1.00	1625	3236

Regression Coefficients:

Estimate Est.Error l-95% CI u-95% CI Rhat Bulk_ESS Tail_ESS

Intercept	4.07	0.12	3.85	4.31 1.00	1422	2335

MixedSpecies	-0.65	0.18	-1.02	-0.29 1.00	1693	2594

OnlyMahi	-0.91	0.19	-1.28	-0.54 1.00	1790	2828

Effect of predator composition on attack interval (3 *only mahi* hunts with sea lions omitted)

Family: gamma

Links: mu = log

Formula: TimebwAttacks_focal ~ Treatment + (1 | ID)

Data: data (Number of observations: 2036)

Draws: 4 chains, each with iter = 4000; warmup = 2000; thin = 1;

total post-warmup draws = 8000

Multilevel Hyperparameters:

~ID (Number of levels: 29)

Estimate Est.Error l-95% CI u-95% CI Rhat Bulk_ESS Tail_ESS

sd(Intercept	0.44	0.07	0.32	0.61 1.00	1820	3132

Regression Coefficients:

Estimate Est.Error l-95% CI u-95% CI Rhat Bulk_ESS Tail_ESS

Intercept	4.07	0.12	3.83	4.32 1.00	1287	2293

MixedSpecies	-0.65	0.20	-1.03	-0.25 1.00	1504	2998

OnlyMahi	-0.96	0.24	-1.43	-0.48 1.00	2125	3436

Effect of predator composition on capture interval:

Family: gamma

Links: mu = log

Formula: CaptureInterval_All_focal ~ Treatment + (1 | ID)

Data: data (Number of observations: 988)

Draws: 4 chains, each with iter = 4000; warmup = 2000; thin = 1;

total post-warmup draws = 8000

Multilevel Hyperparameters:

~ID (Number of levels: 29)

Estimate Est.Error l-95% CI u-95% CI Rhat Bulk_ESS Tail_ESS

sd(Intercept)	0.68	0.12	0.49	0.94 1.00	2031	3655

Regression Coefficients:

Estimate Est.Error l-95% CI u-95% CI Rhat Bulk_ESS Tail_ESS

Intercept	4.91	0.19	4.53	5.29 1.01	1305	2191

MixedSpecies	-0.91	0.31	-1.52	-0.30 1.00	1659	2998

OnlyMahi	-1.09	0.40	-1.86	-0.30 1.00	2028	3781

Effect of predator composition on isolation interval:

Family: gamma

Links: mu = log

Formula: IsolationInterval_focal ~ Treatment + (1 | ID)

Data: data (Number of observations: 530)

Draws: 4 chains, each with iter = 4000; warmup = 2000; thin = 1;

total post-warmup draws = 8000

Multilevel Hyperparameters:

~ID (Number of levels: 29)

Estimate Est.Error l-95% CI u-95% CI Rhat Bulk_ESS Tail_ESS

sd(Intercept)	0.78	0.17	0.50	1.17 1.00	1922	3857

Regression Coefficients:

Estimate Est.Error l-95% CI u-95% CI Rhat Bulk_ESS Tail_ESS

Intercept	5.45	0.23	4.99	5.91 1.00	1643	2770

TreatmentB	-1.10	0.37	-1.82	-0.36 1.00	2126	3435

TreatmentC	-0.92	0.55	-2.03	0.15 1.00	2799	3776

**Capture Efficiencies**

The effect of predator composition on capture efficiencies:

Family: beta

Links: mu = logit; phi = identity

Formula: capture_efficiency ~ Treatment

Data: data_cap_eff_trans (Number of observations: 37)

Draws: 4 chains, each with iter = 4000; warmup = 2000; thin = 1;

total post-warmup draws = 8000

Regression Coefficients:

Estimate Est.Error l-95% CI u-95% CI Rhat Bulk_ESS Tail_ESS

Intercept	-1.35	0.19	-1.73	-0.98 1.00	5702	5129

MarlinMixed	-0.40	0.32	-1.04	0.21 1.00	6046	5585

MahiMixed	1.12	0.26	0.61	1.64 1.00	6103	5476

OnlyMahi	0.48	0.32	-0.14	1.09 1.00	6362	5634

**Nature of Interactions between striped marlin and mahi-mahi**

Effect of mahi-mahi presence on striped marlin attack interval:

Family: gamma

Links: mu = log

Formula: TimebwAttacks_smonly_focal ~ Treatment + (1 | ID)

Data: data_AB (Number of observations: 1650)

Draws: 4 chains, each with iter = 4000; warmup = 1000; thin = 1;

total post-warmup draws = 12000

Multilevel Hyperparameters:

~ID (Number of levels: 21)

Estimate Est.Error l-95% CI u-95% CI Rhat Bulk_ESS Tail_ESS

sd(Intercept)	0.55	0.11	0.38	0.79 1.00	2409	3911

Regression Coefficients:

Estimate Est.Error l-95% CI u-95% CI Rhat Bulk_ESS Tail_ESS

Intercept	4.04	0.16	3.73	4.35 1.00	2086	3432

MarlinMixed	-0.26	0.26	-0.79	0.25 1.00	2701	4111

Effect of mahi-mahi presence on striped marlin capture interval:

Family: gamma

Links: mu = log

Formula: CaptureInterval_w_isol_smonly_focal ~ Treatment + (1 | ID)

Data: data_AB (Number of observations: 792)

Draws: 4 chains, each with iter = 4000; warmup = 2000; thin = 1;

total post-warmup draws = 8000

Multilevel Hyperparameters:

~ID (Number of levels: 21)

Estimate Est.Error l-95% CI u-95% CI Rhat Bulk_ESS Tail_ESS

sd(Intercept)	0.71	0.15	0.48	1.04 1.00	1386	2694

Regression Coefficients:

Estimate Est.Error l-95% CI u-95% CI Rhat Bulk_ESS Tail_ESS

Intercept	4.91	0.21	4.49	5.32 1.01	1129	1876

MarlinMixed	-1.07	0.36	-1.78	-0.36 1.00	1687	2874

Effect of mahi-mahi presence on proportion of striped marlin captures that are isolates:

Family: bernoulli

Links: mu = logit

Formula: Isolated ~ Treatment + (1 | ID)

Data: data_AB_captures (Number of observations: 818)

Draws: 4 chains, each with iter = 4000; warmup = 2000; thin = 1;

total post-warmup draws = 8000

Multilevel Hyperparameters:

~ID (Number of levels: 22)

Estimate Est.Error l-95% CI u-95% CI Rhat Bulk_ESS Tail_ESS

sd(Intercept)	0.44	0.21	0.05	0.89 1.00	1540	1408

Regression Coefficients:

Estimate Est.Error l-95% CI u-95% CI Rhat Bulk_ESS Tail_ESS

Intercept	0.25	0.16	-0.07	0.58 1.00	2742	3187

MarlinMixed	1.53	0.42	0.74	2.38 1.00	5560	5448

Effect of striped marlin presence on mahi-mahi attack interval:

Family: gamma

Links: mu = log

Formula: TimebwAttacks_mmonly_focal ~ Treatment + (1 | ID)

Data: data_BC (Number of observations: 750)

Draws: 4 chains, each with iter = 4000; warmup = 2000; thin = 1;

total post-warmup draws = 8000

Multilevel Hyperparameters:

~ID (Number of levels: 19)

Estimate Est.Error l-95% CI u-95% CI Rhat Bulk_ESS Tail_ESS

sd(Intercept)	0.47	0.12	0.29	0.75 1.00	2346	3731

Regression Coefficients:

Estimate Est.Error l-95% CI u-95% CI Rhat Bulk_ESS Tail_ESS

Intercept	3.09	0.18	2.74	3.44 1.00	2430	3496

MahiMixed	0.08	0.25	-0.41	0.58 1.00	2211	2608

Effect of striped marlin presence on mahi-mahi attack interval (3 *only mahi* hunts with sea lions omitted):

Family: gamma

Links: mu = log

Formula: TimebwAttacks_mmonly_focal ~ Treatment + (1 | ID)

Data: data_BC (Number of observations: 343)

Draws: 4 chains, each with iter = 4000; warmup = 2000; thin = 1;

total post-warmup draws = 8000

Multilevel Hyperparameters:

~ID (Number of levels: 16)

Estimate Est.Error l-95% CI u-95% CI Rhat Bulk_ESS Tail_ESS

sd(Intercept)	0.49	0.15	0.25	0.83 1.00	2192	3717

Regression Coefficients:

Estimate Est.Error l-95% CI u-95% CI Rhat Bulk_ESS Tail_ESS

Intercept	3.13	0.19	2.75	3.49 1.00	2741	3480

MahiMixed	0.02	0.31	-0.56	0.66 1.00	2885	3790

Effect of striped marlin presence on mahi-mahi capture interval:

Family: gamma

Links: mu = log

Formula: CaptureInterval_w_isol_mmonly_focal ~ Treatment + (1 | ID)

Data: data_BC (Number of observations: 179)

Draws: 3 chains, each with iter = 3000; warmup = 1500; thin = 1;

total post-warmup draws = 4500

Multilevel Hyperparameters:

~ID (Number of levels: 16)

Estimate Est.Error l-95% CI u-95% CI Rhat Bulk_ESS Tail_ESS

sd(Intercept)	0.64	0.20	0.32	1.11 1.00	1288	2208

Regression Coefficients:

Estimate Est.Error l-95% CI u-95% CI Rhat Bulk_ESS Tail_ESS

Intercept	3.71	0.25	3.20	4.19 1.00	1358	2255

MahiMixed	0.27	0.41	-0.53	1.08 1.00	1614	2445

Effect of striped marlin presence on proportion of mahi-mahi captures that are isolates:

Family: bernoulli

Links: mu = logit

Formula: Isolated ~ Treatment + (1 | ID)

Data: data_BC_captures (Number of observations: 201)

Draws: 4 chains, each with iter = 4000; warmup = 2000; thin = 1;

total post-warmup draws = 8000

Multilevel Hyperparameters:

~ID (Number of levels: 16)

Estimate Est.Error l-95% CI u-95% CI Rhat Bulk_ESS Tail_ESS

sd(Intercept)	0.29	0.21	0.01	0.80 1.00	2874	3327

Regression Coefficients:

Estimate Est.Error l-95% CI u-95% CI Rhat Bulk_ESS Tail_ESS

Intercept	-0.59	0.23	-1.06	-0.16 1.00	6195	4342

MahiMixed	0.46	0.39	-0.31	1.20 1.00	6170	4264

**Transitional Probabilities (corrected for group size)**

glm(formula = next_is_sm ~ Behaviour + offset(logit_exp_sm),

family = binomial(), data = attack_transitions_model)

Coefficients:

Estimate Std. Error z value	Pr(>|z|)

(Intercept)	-0.3065	0.2982 -1.028 0.30398

Behaviourmm attack -1.0905	0.3788 -2.879 0.00399 **

**Spatial analysis**

The effect of species on normalized Voronoi area:

Family: gamma

Links: mu = log; shape = identity

Formula: norm_voronoi_area ~ species + (1 | ID)

Data: data2 (Number of observations: 744)

Draws: 4 chains, each with iter = 4000; warmup = 2000; thin = 1;

total post-warmup draws = 8000

Multilevel Hyperparameters:

~ID (Number of levels: 26)

Estimate Est.Error l-95% CI u-95% CI Rhat Bulk_ESS Tail_ESS

sd(Intercept)	0.47	0.08	0.34	0.66 1.00	2328	3317

Regression Coefficients:

Estimate Est.Error l-95% CI u-95% CI Rhat Bulk_ESS Tail_ESS

Intercept	-1.99	0.12	-2.23	-1.75 1.00	1938	3062

speciesMarlin	0.55	0.10	0.35	0.76 1.00	5605	5876

The effect of species and time on predator distance to the prey school pre- and post- attack:

Family: gamma

Links: mu = log; shape = identity

Formula: Distance_pos ~ Species * factor(Time) + (1 | ID/AttackFrame)

Data: data (Number of observations: 514)

Draws: 4 chains, each with iter = 4000; warmup = 2000; thin = 1;

total post-warmup draws = 8000

Multilevel Hyperparameters:

~ID (Number of levels: 6)

Estimate Est.Error l-95% CI u-95% CI Rhat Bulk_ESS Tail_ESS

sd(Intercept)	0.55	0.34	0.08	1.40 1.00	2276	2006

~ID:AttackFrame (Number of levels: 25)

Estimate Est.Error l-95% CI u-95% CI Rhat Bulk_ESS Tail_ESS

sd(Intercept)	0.59	0.13	0.38	0.89 1.00	3231	4926

Regression Coefficients:

Estimate Est.Error l-95% CI u-95% CI Rhat Bulk_ESS Tail_ESS

Intercept	5.85	0.49	4.92	6.87 1.00	1353	2488

SpeciesMarlin	0.11	0.58	-1.02	1.23 1.01	1010	2042

factorTimeM4.5	-0.13	0.52	-1.15	0.89 1.00	1694	2900

factorTimeM4	-0.20	0.53	-1.24	0.83 1.00	1594	2914

factorTimeM3.5	-0.54	0.53	-1.56	0.51 1.00	1396	2869

factorTimeM3	-0.71	0.52	-1.72	0.34 1.00	1313	2778

factorTimeM2.5	-0.74	0.53	-1.77	0.28 1.00	1521	2963

factorTimeM2	-0.76	0.52	-1.79	0.29 1.00	1554	2414

factorTimeM1.5	-0.88	0.53	-1.92	0.15 1.00	1651	2503

factorTimeM1	-1.15	0.53	-2.18	-0.11 1.00	1584	2774

factorTimeM0.5	-1.54	0.54	-2.59	-0.48 1.00	1520	2762

factorTime0	-4.42	0.54	-5.47	-3.36 1.00	1572	2691

factorTime0.5	-1.83	0.53	-2.86	-0.80 1.00	1437	2817

factorTime1	-1.44	0.54	-2.50	-0.37 1.00	1857	2453

factorTime1.5	-1.51	0.53	-2.54	-0.47 1.00	1330	2557

factorTime2	-1.64	0.53	-2.67	-0.56 1.00	1386	2806

factorTime2.5	-1.40	0.53	-2.44	-0.33 1.00	1684	2822

factorTime3	-1.17	0.53	-2.21	-0.14 1.00	1681	2864

factorTime3.5	-1.15	0.54	-2.20	-0.10 1.00	1418	2751

factorTime4	-1.04	0.54	-2.08	0.00 1.00	1374	2542

factorTime4.5	-1.00	0.53	-2.03	0.00 1.00	1640	2562

factorTime5	-0.99	0.53	-2.03	0.05 1.00	1619	2441

SpeciesMarlin:factorTimeM4.5	0.28	0.72	-1.15	1.72 1.00	1512	3474

SpeciesMarlin:factorTimeM4	0.33	0.73	-1.13	1.75 1.00	1531	3410

SpeciesMarlin:factorTimeM3.5	0.56	0.71	-0.83	1.95 1.00	1465	3212

SpeciesMarlin:factorTimeM3	0.56	0.72	-0.84	1.97 1.00	1274	3343

SpeciesMarlin:factorTimeM2.5	0.32	0.72	-1.08	1.72 1.00	1441	3258

SpeciesMarlin:factorTimeM2	0.06	0.71	-1.35	1.44 1.00	1453	3220

SpeciesMarlin:factorTimeM1.5	-0.07	0.71	-1.47	1.33 1.00	1422	3045

SpeciesMarlin:factorTimeM1	-0.31	0.72	-1.73	1.09 1.00	1418	3260

SpeciesMarlin:factorTimeM0.5	-0.97	0.74	-2.43	0.47 1.00	1538	3433

SpeciesMarlin:factorTime0	-1.14	0.73	-2.57	0.30 1.00	1382	3000

SpeciesMarlin:factorTime0.5	-2.27	0.73	-3.69	-0.84 1.00	1517	3433

SpeciesMarlin:factorTime1	-1.01	0.75	-2.50	0.48 1.00	1485	3033

SpeciesMarlin:factorTime1.5	-0.28	0.72	-1.71	1.15 1.00	1286	3315

SpeciesMarlin:factorTime2	0.08	0.73	-1.35	1.50 1.00	1426	3205

SpeciesMarlin:factorTime2.5	0.47	0.73	-1.00	1.89 1.00	1611	3517

SpeciesMarlin:factorTime3	0.65	0.72	-0.75	2.07 1.00	1397	2928

SpeciesMarlin:factorTime3.5	1.02	0.72	-0.40	2.47 1.00	1474	3081

SpeciesMarlin:factorTime4	0.98	0.73	-0.44	2.42 1.00	1378	3059

SpeciesMarlin:factorTime4.5	1.03	0.72	-0.39	2.42 1.00	1365	3103

SpeciesMarlin:factorTime5	1.11	0.73	-0.32	2.53 1.00	1494	3251

Bibliography

1.	Pacher, K. *et al.* Evidence for a by‐product mutualism in a group hunter depends on prey movement state. *Funct. Ecol.* **38**, 2123–2138 (2024).

2.	Hansen, M. J. *et al.* California sea lions interfere with striped marlin hunting behaviour in multi-species predator aggregations. *Philos. Trans. R. Soc. B Biol. Sci.* (2023).

3.	Friard, O. & Gamba, M. BORIS: a free, versatile open‐source event‐logging software for video/audio coding and live observations. *Methods Ecol. Evol.* **7**, 1325–1330 (2016).

References included in revision

Bideau, P., Pham, D., Dhellemmes, F., Hansen, M., & Krause, J. (2026). Watching Swarm Dynamics from Above: A Framework for Advanced Object Tracking in Drone Videos. *International Journal of Computer Vision*.

Fonseca, S., Milho, J., Travassos, B., & Araújo, D. (2012). Spatial dynamics of team sports exposed by Voronoi diagrams. *Human movement science*, *31*(6), 1652-1659.

Raabe, D., Biermann, H., Bassek, M., Memmert, D., & Rein, R. (2024). The dual problem of space: Relative player positioning determines attacking success in elite men’s football. *Journal of Sports Sciences*, *42*(19), 1821-1830.

Kim, S. (2004). Voronoi analysis of a soccer game. *Nonlinear Analysis: Modelling and Control*, *9*(3), 233-240.
